# The efficacy of acupuncture and decompression splints in the treatment of temporomandibular joint pain-dysfunction syndrome

**DOI:** 10.4317/medoral.17567

**Published:** 2012-05-01

**Authors:** Mario Vicente-Barrero, Si-Lei Yu-Lu, Bingxin Zhang, Sacramento Bocanegra-Pérez, David Durán-Moreno, Adriana López-Márquez, Milan Knezevic, José-María Castellano-Navarro, José-María Limiñana-Cañal

**Affiliations:** 1 MD, DDS, PhD, Doctor in Medicine. Specialized in Stomatology. Associate Professor at the University of Las Palmas, Grand Canary Island (Universidad de Las Palmas de Gran Canaria); 2 Physician. Acupuncturist; 3 Graduated in Traditional Chinese Medicine; 4 Doctor in medicine. Specialized in Stomatology; 5 Physician, specialized in Oral and Maxillofacial Surgery; 6 Dentist; 7 Doctor in Medicine. Specialized in Oral and Maxillofacial Surgery; 8 Physician and Dentist; 9 Research Department. Professor of Statistics at the University of Las Palmas, Grand Canary Island (Universidad de Las Palmas de Gran Canaria)

## Abstract

Objectives: The goal of the present study was to evaluate the results of applying acupuncture or occlusal decompression splints in the treatment of patients diagnosed with the temporomandibular joint pain-dysfunction syndrome. Design of the study: We conducted a randomized clinical trial including 20 patients to whom the mentioned treatments were applied. Results were evaluated through an analogue pain scale, measurements of mouth opening and jaw lateral deviation in millimetres, and assessment of sensitivity to pressure on different points: preauricular, masseter muscle, temporal muscle and trapezius. Parameters were evaluated before and 30 days after the treatment. For standardized pressure, we used a pressure algometer. Results: Patients treated with decompression splints showed reductions in subjective pain and pain upon pressure on temporal, masseter and trapezius muscles, as well as increased mouth opening after the treatment. Patients treated with acupuncture showed pain reduction in the short term and improvements in all of the evaluated para-meters (stronger pressure was required to produce pain; mouth opening was improved). Conclusion: Acupuncture was an effective complement and/or an acceptable alternative to decompression splints in the treatment of myofascial pain and temporomandibular joint pain-dysfunction syndrome.

** Key words:**Temporomandibular joint, temporomandibular dysfunction, acupuncture, decompression splint, arthralgia, myofascial pain, joint palpation.

## Introduction

The term “temporomandibular disorders” was suggested by the American Dental Association as a common label for different clinical conditions involving chronic orofacial pain (muscle pain associated to structural components, degenerative disease, myofascial pain-dysfunction syndrome, etc.) (1). Differential diagnosis of subtypes is difficult. Signs and symptoms corresponding to different types may overlap and change spontaneously over the time.

Myofascial pain, also called myofascial pain-dysfunction syndrome (PDS) is the most frequent subtype of temporomandibular disorder, affecting subjects in every socioeconomic and ethnic level (2). The aetiology of this condition seems to be multifactorial. Although symptoms may be variable, one of the following signs and/or symptoms will be present: pain in temporomandibular joint, pain upon palpation of associated muscles, restriction or deviation of mandible movement, joint noise and headache. There is no standard treatment for reducing myofascial pain and many patients are refractive to treatment. Thus, a number of different therapies, isolated or combined, have been used (pharmacological, occlusal, psychotherapeutic, physiotherapeutic treatments, etc.) (2).

Pain relief is the main goal of such treatments (3). Usually, the first therapeutic approach is pharmacological (antidepressants, benzodiazepines, muscle relaxants, non-opioid analgesics). However, the efficacy of such drugs is not confirmed and the possible side effects derived from long-term administration need to be seriously consi-dered. Moreover, the natural history of orofacial pain, with treatment-independent remissions and exacerbations, may also influence the outcome of drug-therapy.

A number of physical techniques have been tried to treat PDS (deep tissue-massage, muscle stretching/relaxation, transcutaneous electrical nerve stimulation, injections on trigger points and use of decompression splints or muscle-relaxation splints). Some of them have been accepted after exhaustive scientific discussion; others lack evidence enough to support their therapeutic effects (4).

Acupuncture originated in China more than 3000 years ago. Beneficial effects of this technique in the management of PDS have been reported (5-8). Although the action mechanism of acupuncture is not fully understood, several explanations have been proposed. It is nowadays accepted that acupuncture stimulates small myelinated nerve fibbers in muscles, which in turn send impulses to the spinal cord, thus stimulating three centres: the spinal cord, the mesencephalon and the hypothalamus-hypophysis axis. Furthermore, it has been demonstrated that several neurotransmitters, such as enkephalins, beta endorphin, dynorphin, serotonin and noradrenalin are involved in this process (9).

Results of controlled clinical trials suggest that the effects of acupuncture therapy are similar to those of stabilization splints (5,10-13).

The goal of this study was to assess the results of applying acupuncture or occlusal decompression splints to treat patients with PDS; as well as to evaluate the efficacy of such treatments.

## Material and Methods

This study included 20 patients, who had visited the Dental Care Services of different Primary Health Centres in the Canary Islands and had been referred to the Department of Stomatology and Oral and Maxillofacial Surgery of the Hospital Insular de Gran Canaria with symptoms compatible with a diagnosis of muscle-related PDS. This study was approved by the Hospital Commission for Research, Teaching and Training.

Patients were selected according to the following inclusion criteria:

• Three-month or longer history of at least two of the following signs or symptoms: pain upon palpation of the temporomandibular joint (TMJ) or associated muscles of mastication, restriction or deviation of jaw movement, headache plus joint noise. Headache and joint noise were not considered when they occurred separately.

• Legal age.

• Normal vertical dimension with complete or almost complete dentition.

Exclusion criteria were.

• Legal involvement such as traffic accidents, sick leave, etc.

• Dental malocclusion with variations from normal vertical dimension.

• Malignancies or other diseases, especially those involving other joints.

• Bone and/or degenerative diseases.

• Headache associated with general organic conditions.

• Fibromyalgia.

• Mental disorders.

• Previous treatment with acupuncture and/or decompression splint.

• Previous surgery of the TMJ.

• Orthodontic treatment at the time of the study.

• Wearing a complete removable prosthesis.

• Allergy to metal.

Patients were randomly allocated to either decompression splint or acupuncture therapy. The studied population consisted of 20 patients (17 females; 3 males) between 18 and 58 years of age (average 39 years); 8 females and 2 males were treated with acupuncture; 9 females and 1 male were treated with decompression splints. All of the enrolled patients signed an informed consent form. All of them underwent.

▪ detailed physical examination and preparation of complete clinical record.

▪ mouth-dental examination to assess tooth-decay, periodontal disease and general mouth hygiene.

▪ specific TMJ examination.

▪ orthopantomography.

Treatments were applied by two operators: a physician specialized in stomatology, who was in charge of patient examination before and after the treatments, as well as of designing the decompression splints; and an acupuncturist graduated in traditional Chinese medicine, who applied the acupuncture treatment to all patients in the acupuncture group.

**Figure 1:**
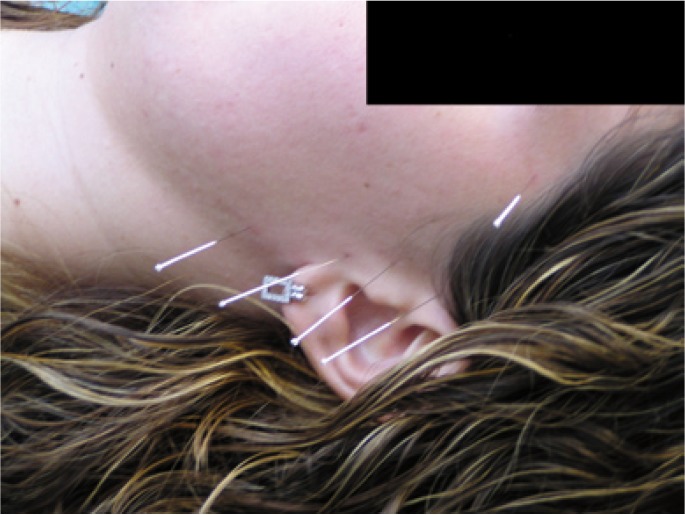
Acupoints EX-HN5, SJ 21, GB2, SJ17, ST 6 in a patient.

Patients with the acupuncture therapy were treated with local and distal acupuncture points (acupoints). Local acupoints were: EX-HN5, SJ 21, GB2, SJ17, ST6 (Fig. 1) and distal acupoints were: LI-4, ST-36, SJ5 and GB34. We used steel 0.25 mm x 25 mm needles, individually labelled for each patient on the first session, disinfected (with alcohol 70% and povidone-iodine) before and after every session.

Needles were inserted 3-5 mm epicutaneous using a device specially designed to reduce patient’s bother and anxiety and were maintained for 30 minutes on every session. The acupuncture treatment consisted of 15 sessions: the first three ones were conducted on consecutive days and the rest of them were conducted on a three-per-week basis for a total of 5 weeks.

Patients treated with decompression splints received decompression splints preferentially on the upper arch, except when upper molars were absent; in that case, the splint was placed on the lower arch. Splints were designed with the following characteristics.

• Stable occlusion with a maximum number of contacts.

• Canine guidance.

• Absence of contacts on the non working side.

• Only overnight use.

None of these patients received concomitant pharmacological treatment with analgesic, anti-inflammatory or muscle-relaxant drugs.

Treatment efficacy was evaluated through:

• Mouth opening and lateral jaw-deviation, measured in millimeters.

• Sensitivity to pressure on certain areas: preauricular, masseter muscle, temporal muscle and trapezius, before and 30 days after the treatment. For standardized pressure we used a pressure algometer (Force Dial™, Wagner Instruments, Greenwich, USA).

• Visual analogue pain scale, where 0 corresponded to absence of pain and 10 corresponded to the worst imaginable pain.

Numerical variables were expressed as mean and standard deviation. Sample normality was evaluated with the non-parametric Kolmogorov-Smirnov test. The pre- and post-treatment mean values were compared by using the Student’s t-test for dependent samples. Hypotheses contrast was considered to be significant for p-values lower than 0.05. We used the SPSS 14.0.1 statistical package.

## Results

Both groups of patients – treated during five weeks – showed reduction of myofascial pain in the short term.

**Figure 2:**
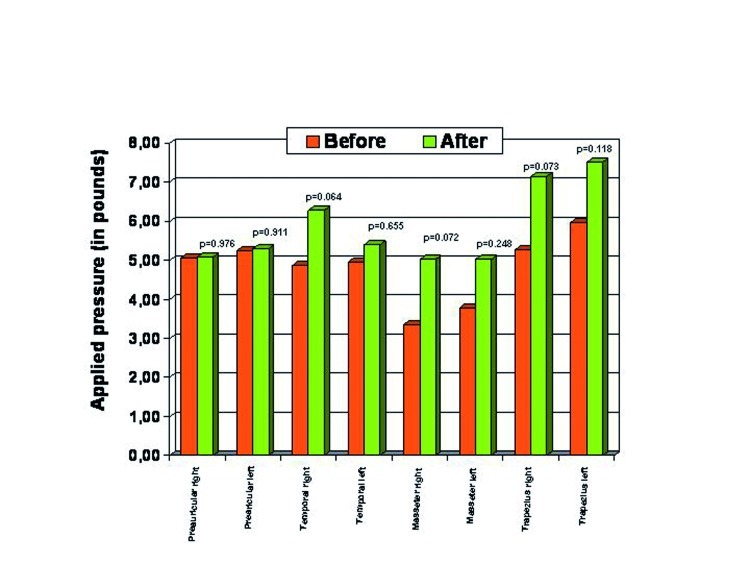
Pressure points before and after the treatment with decompression splint.

Patients treated with decompression splints showed reductions in subjective pain and pain on pressure points located on the temporal, masseter and trapezius muscles, although differences did not reach statistical significance (p>0.05) (Fig. 2).

**Figure 3:**
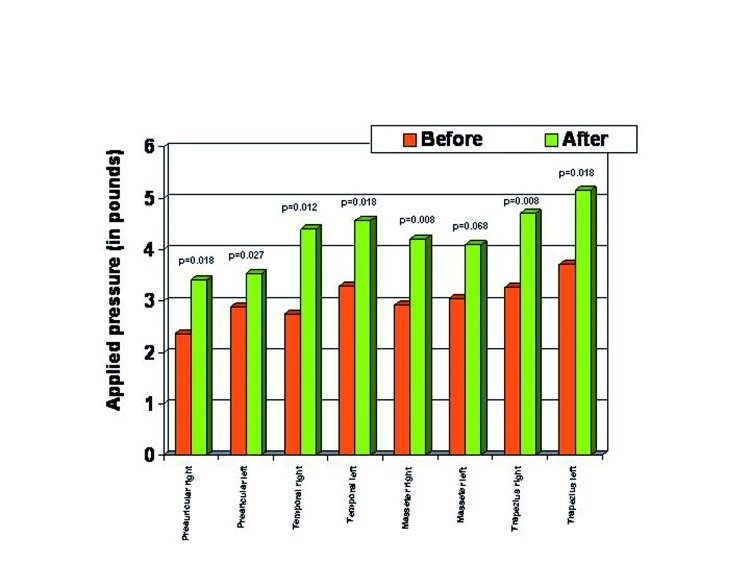
Pressure points before and after the treatment with acupuncture.

Patients treated with acupuncture experienced significant improvements in all of the studied parameters (reduced subjective pain, stronger algometer pressure needed to produce pain, mouth opening improved). Pain reduction was statistically significant (p<0.05) for all the evaluated points except the one located on the masseter muscle (p=0.068) (Fig. 3).

Since we did not conduct long-term patient follow-up these results are restricted to immediate effects of both treatments. Patients who had received a decompression splint were recommended to keep on wearing it on a regular basis.

## Discussion

Acupuncture is currently considered an effective therapeutic alternative for most clinical conditions involving chronic pain (9,14,15). Cho et al. (16) recently published a systematic review and verified the efficacy of acupuncture in reducing PDS-associated symptoms. Ritenbaugh et al. (17) also reported good results with an acupuncture treatment of PDS.

The two most frequent conditions treated with acupuncture are headache and low back pain, although this therapy has also been used to treat sore throat, post-surgical dental pain, oncologic pain, polyneuritis, rheumatoid arthritis, sciatica, bursitis, tendinitis, etc. (18).

Acupuncture is currently considered to be effective to treat dental pain, as well as postoperative and post-chemotherapy nausea and vomiting. Positive results have been reported with migraine, low back pain and temporomandibular disorders. Some authors consider that results are positive, while others consider them difficult to interpret. In general, this therapy is considered a relatively safe procedure (19). Published reports from high-quality research work evaluate acupuncture as a positive treatment for PDS (5-7,10-13).

The prevalence of PDS is equivalent among both men and women. Women however, demand treatment more often; 85% of our subjects were women.

Coincidently with other authors (2,8) we observed reduced myofascial pain in the short term (5 weeks), for all patients treated with acupuncture.

Few studies document long term results of acupuncture or interocclusal splints. List and Helkimo (13) concluded in a follow-up study, that most patients still exhibited improvement of their symptoms one year after treatment. In another long-term study, Bergström et al. (20) examined 55 patients, who had received acupuncture and/or interocclusal splint 18-20 years before, and found long lasting symptom improvement for most of them. Furthermore, more than a quarter of all the patients treated with interocclusal splint were still wearing it regularly. However, the results of that study were rather subjective, based on a postal questionnaire. Other studies, more similar to the present one, evaluate the treatment results in the short term. Johansson et al. (6) studied 45 patients with long-standing headache of muscular origin and compared the results of an acupuncture treatment with those of muscle-relaxation splints. Patients were examined before the treatment and 3 months after the treatment. Patients had to self-evaluate their pain as: nonexistent, mild, moderate, severe and very severe, by using a visual analogue scale. Clinical examination of the stomatognathic system included palpation of masticatory muscles, joint noise, joint movement, pain upon jaw movement, deviation from midline during mouth opening and evaluation of occlusal interferences. No significant differences were found between treatments; thus, the authors concluded that acupuncture was a valid alternative to conventional treatments. Even when the mentioned study was well-designed, we consider that results were highly subjective, both those self-evaluated by the patient and those evaluated by the examiner. In particular, we believe that item “pain upon palpation” should not have been considered an objective factor, since pressure could have been rather variable. Therefore, we postulate that a pressure algometer should be used in this type of studies, so as to produce comparable results.

Occlusal splints are extensively used to treat PDS. Several hypotheses have been proposed to explain their effectiveness (21-25), all of them though, based on the assumption that splints have a real therapeutic value (26). In this study, we compared the therapeutic effects of acupuncture with those of occlusal splints, in the treatment of PDS.

As mentioned, reliable measurement of pain upon palpation of TMJ and associated muscles entails major difficulties, derived from inter- and intra-observer variability, as well as from variations of subjective pain reported by patients at different times (27). In order to minimize such errors, Goulet and Clark (28) proposed the use of a pressure algometer. These authors demonstrated that the pain threshold upon pressure on masticatory muscles can be more reliably measured with an algometer than through palpation. Accordingly, Fernández-Carnero et al. (29) used an electronic algometer to assess pain threshold upon pressure. Their results support the hypothesis that acupuncture may have beneficial effects on PDS signs and symptoms at least in the short term. On this basis, we also used a pressure algometer in this study, so as to prevent examiner variability.

Raustia et al. (10-12) studied 50 patients randomly divided into two groups. The only criterion for recruiting patients was that they had a complete set of teeth. Clinical examination included measurement of mouth opening, palpation of the TMJ and masticatory muscles and assessment of pain upon joint movement. One group was treated with acupuncture and one group, with a standard stomatognathic treatment: occlusal adjustment, physiotherapy, decompression splints or a combination of these. Acupoints were selected for each patient in particular, on the basis of previous clinical examination and were applied in 3 sessions of 20 minutes each. Patients were examined after 1 week of treatment and 3 months after the treatment. No statistically significant differences between treatments were found. Like in the above study by Johansson et al. (6) pressure upon palpation was not objectively measured. Furthermore, the authors used combinations of both treatments and acupoints were different for different patients. Actually, because of the characteristics of acupuncture, it is difficult to follow a standardized treatment protocol. However, in this study, we were able to use only one combination of acupoints.

Finally, we would like to consider a possible placebo effect of acupuncture. Goddar (8) presented a comparative study of the effects of actual acupuncture with sham acupuncture and observed very positive effects on TMJ-associated signs and symptoms in the short term. In a recent randomized trial, Shen et al. (30) demonstrated the effectiveness of real versus sham acupuncture to treat temporomandibular muscle pain, even after one session.

Because of time restrictions, patients could not be followed during a longer period and a larger sample of subjects could not be recruited. Longer time and more subjects could have produced better and more conclusive results.

The results of this study indicate that acupuncture has analgesic effects in the short-term and is therefore comparably effective to occlusal splints, in the treatment of TMJ-PDS.
